# Supplementary material for: Effects of music advertised to support focus on mood and processing speed
Source: PLoS One. 2025 Feb 12;20(2):e0316047. doi: 10.1371/journal.pone.0316047 (PMC11819607; doi:10.1371/journal.pone.0316047)
Supplement: S1 File — (PDF) [file pone.0316047.s001.pdf]

“Effects of Music Advertised to Support Focus on Mood and Processing Speed

J. Orpella†, D.L. Bowling†, C., Tomaino, & P. Ripollés*.

Supplementary Material

Supplementary Text

Brief descriptions of each of the 23 musical features are listed below along with the MIRtoolbox / Matlab function calls used to extract them. Unless otherwise specified, consult the MIRtoolbox manual for details on the operation of MIRtoolbox function.

Tempo. Estimated number of beats per minute. Due to discrepancies between the MIRtoolbox function 'mirtempo.m' and the perception of tempo by trained musicians (Lange & Frieler, 2018), tempo was determined manually by DB via finger tapping in time with the perceived beat using the software application "Tap That Tempo" (Version 2.1.0; Sander, N. Retrieved from the Apple App Store on October 20, 2021). Tapped tempo values were confirmed by consulting the original composer (for work flow tracks) or songbpm.com (for deep focus and pop hits tracks). This approach conforms to published recommendations (Lange & Frieler, 2018).

Pulse Clarity. Index of the perceived strength of the rhythmic pulse (Lartillot et al. 2008). Function calls:

```
pulseClarity = mirpulseclarity(a); % a is the miraudio object containing the audio data.
pulseClarity_value = mirgetdata(pulseClarity)
```

Fluctuation Entropy. Shannon entropy of the summarized fluctuation spectrum. The summarized fluctuation spectrum describes periodic variations in amplitude across different frequency bands over time. The Shannon entropy of the summarized fluctuation spectrum is an index of rhythmic complexity, with higher values indicated greater complexity. Function calls:

```
fluctuation = mirfluctuation(a,'Mel','Summary');
fluctuationEntropy = mirentropy(fluctuation);
fluctuationEntropy_value = mirgetdata(fluctuationEntropy)
```

Fluctuation Maximum. Ordinate of the peak value of the summarized fluctuation spectrum. An index of the strength of the most dominant rhythmic pattern. Function calls:

```
fluctuation = mirfluctuation(a,'Mel','Summary');
fluctuation_data = mirgetdata(fluctuation);
fluctuationMax_value = max(fluctuationData)
```

Key. Estimated key signature. Values in Table 1 correspond to the percentage of key signatures in each audio conditions estimated to belong to the major mode. Function call:

```
key = mirkey(a)
```

Key Clarity. An index of how strongly the pitch distribution implies a specific key, among all major and minor keys in the chromatic scale. Function calls:

```
[key, keyClarity] = mirkey(a);
keyClarity_value = mirgetdata(keyClarity)
```

Mode. An index of how strongly the pitch distribution implies a major or minor mode. The closer a value is to +1 the more a major mode is implied; the closer a value is to -1 the more a minor mode is implied. Function calls:

```
mode = mirmode;
mode_value = mirgetdata(mode)
```

Chromatic Complexity. The number of prominent chromatic pitch classes, defined as those classes occurring at rates above 50% of the rate of the most frequently occurring pitch class. An index of melodic complexity. Function calls:

```
chromagram = mirchromagram(a);
chromagram_data = mirgetdata(chromagram);
prominentPitchClasses = chromagram_data(chromagram_data>0.5);
chromaticComplexity_value = length(prominentPitchClasses)
```

HCDF Mean. The Harmonic Change Density Function (HCDF) provides an estimate of points of harmonic change over time. The mean of the HCDF is an index harmonic complexity. Function calls:

```
HCDF = mirhcdf(a);
HCDF_data = mirgetdata(HCDF);
HCDF_value = nanmean(HCDF_data)
```

Spectral Flux. Variation in spectral content over time. An index of spectral complexity. Function calls:

```
spectralFlux = mirflux(a);
spectralFlux_data = mirgetdata(spectralFlux);
spectralFlux_value = mean(spectralFlux_data)
```

Spectral Entropy. Shannon entropy of the spectrum. An index of spectral complexity. Function calls:

```
spectralEntropy = mirentropy(a, 'Center');
spectralEntropy_data = mirgetdata(spectralEntropy);
spectralEntropy_value = mean(spectralEntropy_data)
```

Spectral Centroid. The geometric center (in Hz) of the frequency spectrum. An index of how spectral energy is distributed. Function calls:

```
spectralCentroid = mircentroid(a);
spectralCentroid_value = mirgetdata(spectralCentroid)
```

Spectral Spread. The standard deviation of the frequency spectrum. An index of how broadly spectral energy is distributed. Function calls:

```
spectralSpread = mirspread(a);
spectralSpread_value = mirgetdata(spectralSpread)
```

Spectral Flatness. An estimate of how tonal or noise-like the spectrum is. Higher values indicate more noise-like spectra; lower values indicate more tone-like spectra (i.e., with energy concentrated at specific frequencies). Function calls:

```
spectralFlatness = mirflatness(a);
spectralFlatness_value = mirgetdata(spectralFlatness);
```

Spectral Roll-Off. The frequency (in Hz) below which 85% of the total energy in concentrated. An index of high frequency energy content. Function calls:

```
spectralRollOff = mirrolloff(a);
spectralRollOff_data = mirgetdata(spectralRollOff);
spectralRollOff_value = mean(spectralRollOff_data)
```

Brightness. The percentage of total energy above 1500 Hz. An index of high frequency energy content. Function calls:

```
brightness = mirbrightness(a);
brightness_value = mirgetdata(brightness)
```

Zero Crossing Rate. The number of times an audio waveform crosses zero amplitude every second. An index of noisiness (higher values indicate more noisiness). Function calls:

```
zeroCrossingRate = mirzerocross(a);
zeroCrossingRate_value = mirgetdata(zeroCrossingRate)
```

Attack Time. The average duration (in seconds) between the onset of estimated sound events and their peak amplitudes. Function calls:

```
e = mirevents(a);
attackTime = mirattacktime(e);
attackTime_data = mirgetdata(attackTime);
attackTime_value = mean(attackTime_data)
```

Attack Slope. The average slope between the onset of estimated sound events and their peak amplitude. Function calls:

```
e = mirevents(a);
attackSlope = mirattackslope(e);
attackSlope_data = mirgetdata(attackSlope);
attackSlope_value = nanmean(attackSlope_data)
```

Decay Time. The average duration (in seconds) between the amplitude peak of estimated sound events and their offsets. Function calls:

```
e = mirevents(a);
decayTime = mirdecaytime(e);
decayTime_data = mirgetdata(decayTime);
decayTime_value = mean(decayTime_data)
```

Decay Slope. The average slope between the amplitude peak of estimated sound events and their offset. Function calls:

```
e = mirevents(a);
decaySlope = mirdecayslope(e);
decaySlope_data = mirgetdata(decaySlope);
decaySlope_value = nanmean(decaySlope_data)
```

Event Density. Number of estimated sound events per second. Function calls:

```
e = mirevents(a);
eventDensity = mireventdensity(e);
eventDensity_value = mirgetdata(eventDensity)
```

RMS Amplitude. Root Mean Square amplitude.

```
rms = mirrms(a);
rms_data = mirgetdata(rms);
rms_value = mean(rms_data)
```

Supplementary Tables

Table S1. *Track names and artists, set arrangements, and musical features for each track in each audio condition.* The cluster analysis was performed in Matlab (Version 2022a) by passing the musical features for each track (except 'Key') as data to the function 'kmeans.m' with K=2. The silhouette analysis was likewise performed in Matlab, passing the data and the cluster IDs returned by kmeans.m to the function 'silhouette.m'. Increasing K to 3 reduced the mean silhouette value over the set from 0.87 to 0.69 indicating that K=2 represents a better solution.

See attached "S1_Table.xlsx".

Table S2. Preferred music genres in the four groups of participants. The number in each cell indicates the number of participants in that group that indicated that a particular genre was their favorite.

Group	Rock	Pop	Classical	Rap	Jazz	Indie	Electronic	Metal	Country	R&B	Latin	Punk
WF	14	10	7	7	2	3	1	1	4	1	0	0
Focus	14	9	6	6	7	1	2	3	1	0	1	0
Hits	20	10	10	2	2	2	1	1	0	0	0	0
Office	13	10	12	4	2	1	1	0	0	3	0	1

Table S3. Parameter estimates with 95% confidence intervals (CI) for the best model explaining RTs: *FlankerCondition + AudioCondition*TrialNumber + BMRQTotal + GOLD-MSI + DASS21 + (1|ID).* For flanker condition the baseline level is Neutral and for Audio it is Office Noise. Significant parameters are marked in bold.

**Parameter**	**Estimate (CI)**
**Flanker Congruent**	-17.4 [-27.0, -7.8]
**Flanker Incongruent**	72.1 [62.3, 81.8]
Audio Focus	-17.3 [-104.1, 69.5]
Audio Pop Hits	-20.5 [-108.5, 67.5]
Audio Work Flow	-34.8 [-121.7, 52.2]
Trial Number	0.4 [0.0, 0.7]
**BMRQ Total**	-6.0 [-10.3, -1.8]
GOLD Training	145.6 [-27.0, 318.3]
DASS21 Depression	-4.0 [-9.1, 1.0]
**DASS21 Anxiety**	18.2 [11.8, 24.7]
DASS21 Stress	-2.7 [-9.8, 4.4]
Audio Focus * TrialNumber	0.3 [-0.3, 0.8]
Audio Pop Hits * TrialNumber	0.01 [-0.6, 0.5]
**Audio Work Flow * TrialNumber**	-0.77 [-1.3, -0.2]

Table S4. Parameter estimates with 95% confidence intervals (CI) for the exploratory model assessing the relationship between RTs and change in mood for the Work Flow group (see Figure 6 in the main text): *RT ~ TrialNumber*TotalPANAS+ (1|ID).* Significant parameters are marked in bold.

**Parameter**	**Estimate (CI)**
Trial Number	0.0 [-0.5, 0.4]
Total PANAS	49.4 [-97.0, 195.8]
**TrialNumber * Total PANAS**	-0.8 [-1.6, -0.1]

Supplementary Figures

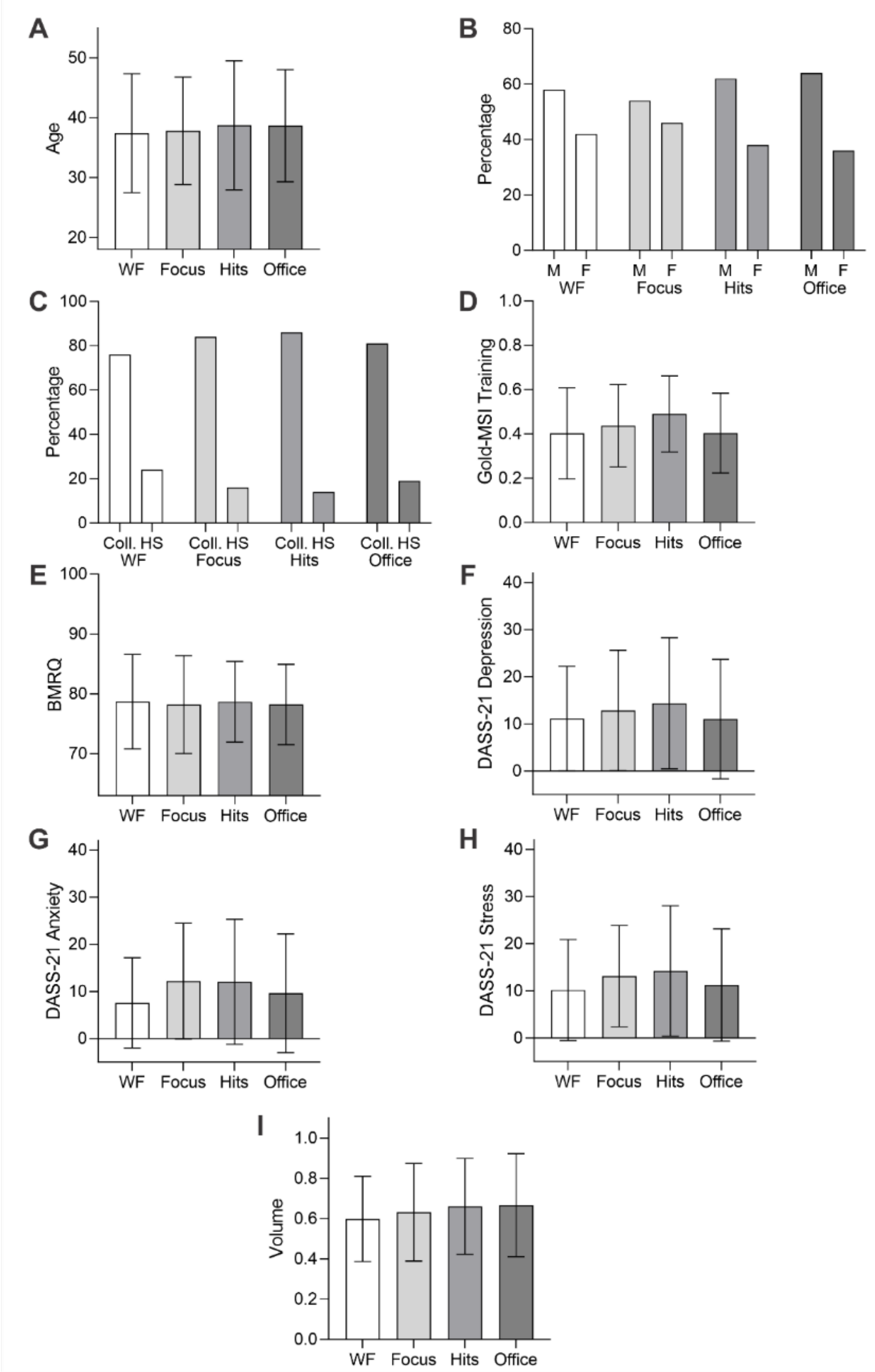

Figure S1. *Group characterization.* Bar plots depicting mean and standard deviation (except for percentages), showing that the four groups of participants who listened to the different audio conditions (work flow [WF], deep focus, pop hits, office noise) were well-balanced in terms of age (**A**), gender (**B**; M: Male, F: Female), education (**C**; Coll: College studies or more, HS: High School Diploma), musical training (**D**), sensitivity to musical reward (**E;** BMRQ Total score), psychological distress (DASS-21: **F,** Depression; **G,** Anxiety; **H**, Stress; each scale varies from 0 to 42), and the volume used during the experiment (**I**; provided using a slider with values ranging from 0 to 1).

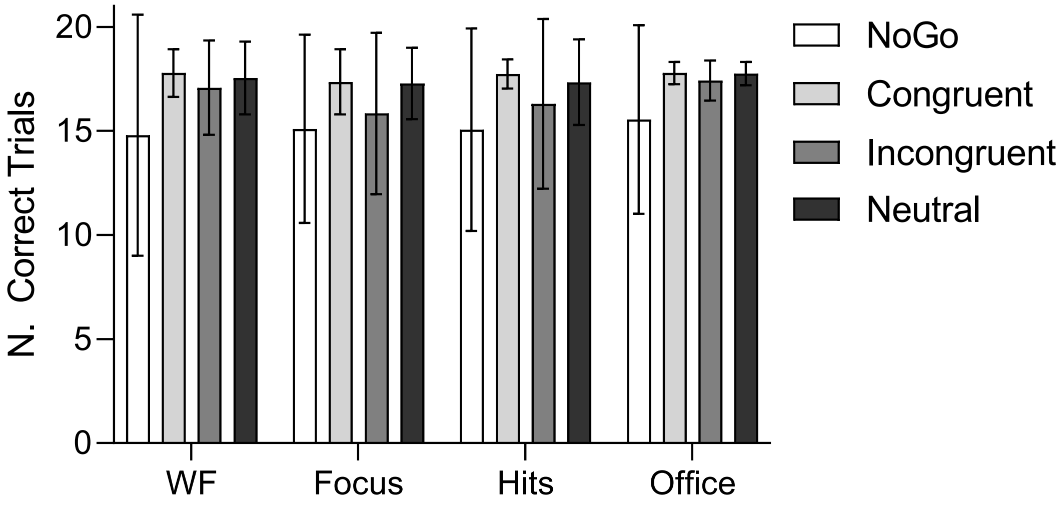

Figure S2. *Accuracy in the flanker task.* Bars show mean and standard deviation for the number of correct trials in each condition in the flanker task (the maximum number of correct trials in all conditions was 18). There was a main effect of the flanker condition, with participants making more errors in the no-go condition as compared with the other conditions, and during the incongruent condition as compared with the neutral and congruent conditions. No main effect of audio condition or significant interaction between audio condition and flanker condition was found. WF = work flow; Focus = deep focus; Hits = pop hits; Office = office noise.

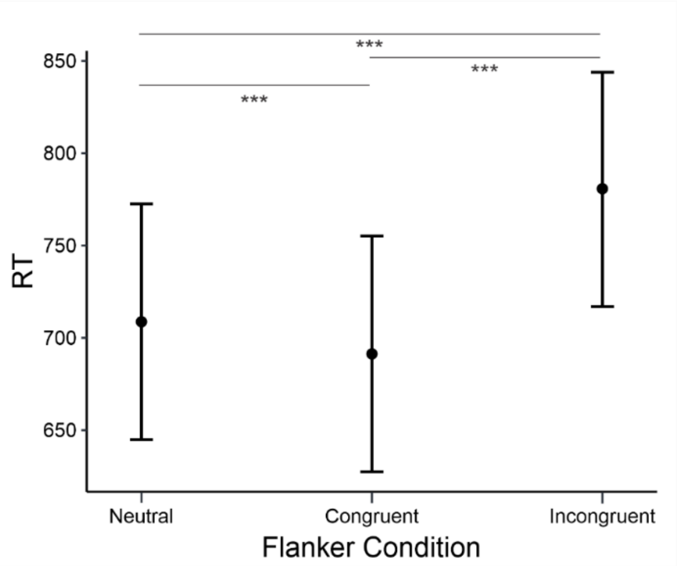

Figure S3. *Main effect of flanker condition on RT.* Predicted effects (with 95% confidence intervals) for the flanker condition fixed factor. *** p<0.001.

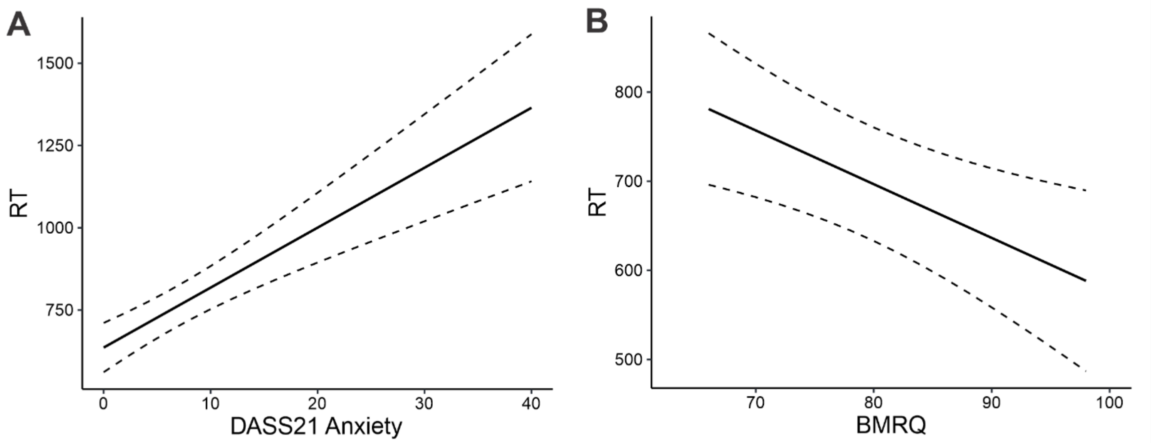

Figure S4. *Main effects of anxiety (A) and sensitivity to musical reward (BMRQ) on RT.* Predicted effects (dashed lines represent 95% confidence intervals) showing that participants who scored higher on the DASS-21 Anxiety scale were slower ($p<0.001$) and participants who scored higher on the BMRQ (i.e., participants with a higher sensitivity to musical reward) were faster during the flanker task ($p=0.005$).

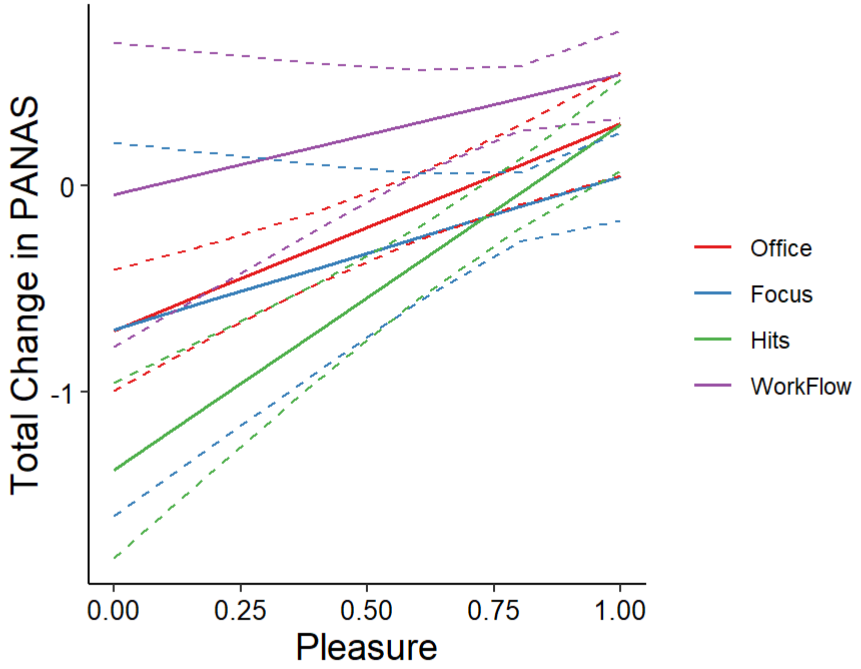

Figure S5. *Effects of perceived pleasure ("liking") on mood.* Exploratory analysis (linear model: Total change in PANAS ~ AudioCondition + Pleasure) showing that the mood improvement induced was modulated by the pleasure the participants felt while listening to each of the audio conditions. Both AudioCondition ($p<0.001$) and Pleasure ($p<0.001$) had significant main effects without an interaction. This suggests that the more pleasure participants felt while listening to any of the tracks, the higher the positive change in mood. The solid lines show predicted values while the dashed lines represent 95% confidence intervals.

Supplementary Audio

Audio S1. Work flow track 1 “You got this” by John Bowers

Audio S2. Work flow track 2 “Pulse if your friend” by John Kunkel

Audio S3. Work flow track 3 “Booster” by Nick Donnelly

Audio S4. Work flow track 4 “Hydration” by Jack Hallenbeck

Audio S5. Office noise, recorded from mynoise.net (see main text for details)
