# Supplementary material for: Effects of music advertised to support focus on mood and processing speed
Source: PLoS One. 2025 Feb 12;20(2):e0316047. doi: 10.1371/journal.pone.0316047 (PMC11819607; doi:10.1371/journal.pone.0316047)
Supplement: Attachment — Submitted filename: 01_AnswerToReviewers_final.docx [file pone.0316047.s010.docx]

We thank the two anonymous reviewers for their helpful and thoughtful comments, which we believe have greatly improved the quality of our manuscript. Below we provide detailed answers to all of the reviewers’ comments and suggestions. Where possible, we have included the edited sections after each comment, and all edited text is printed in blue in our revised manuscript.

**Reviewer #1: ### Summary of paper**

**The current paper evaluates the effects of music advertised to support attentional focus on mood and performance of participants on a cognitively demanding task (the flanker test). The study stands out from previous work as it focuses on evaluating the effect of music in task performance. It is also one of the few studies where the effect is regarding music that is being listened to while the task is being performed (instead of before). The study also investigates the effect of the music in mood and the liking of it by the participants, in order to evaluate possible mechanisms through which changes in task performance may be explained (in particular, the arousal-mood theory). In a between-subjects design, they compared two music sets advertised to improve performance (”work flow” and “deep focus”) with two control conditions: popular music and office background noise. Groups were assessed to be comparable in terms of basic demographic, music-related, and mental health variables. Results showed that only the “work flow” music yielded performance improvement as decreased RT. Music condition had no effect on task accuracy. The paper clearly states the set of a priori statistic analyses by introducing them in the methods section and then exploratory analyses introduced during the result section. A priori analyses were: similarity of participant groups, effect of audio condition on mood change, effect of audio condition on performance accuracy, and effect of audio condition on performance speed considering sensitivity to musical reward, music training and basal psychological distress. Results showed a main effect of anxiety and sensitivity to musical reward for RT as well as decreased RT for the “Work flow” condition. Exploratory analyses focused on looking for possible modulators of the performance improvement effect. Tests were performed for the effect of basal levels of depression, anxiety and stress as well as mood change. Basal levels of depression, anxiety and stress did not show an effect on performance improvement, but mood change did. Greater mood change correlated with lower RTs over time. Additionally, they verified that changes in mood were not predicted by an interaction of music condition and music familiarity or pleasure. Yet, changes in mood were related to musical pleasure. These results are used to hypothesize that the speed improvement in the task may be due to the music’s ability to upregulate arousal and positive affect simultaneously, which seemed to happen mainly with “work flow” music, as it yielded high pleasure as well as high groove ratings, contrasting other music conditions. The conclusions seem to be well supported by the results and the analysis. The three supplementary tables contain all the raw data mentioned in the paper (acoustic features of the stimuli, background data of the participants and individual trial responses).**

We thank the reviewer for their positive and through summary of our work.

**### Main comments**

**Here I detail main concerns regarding presentation.**

**1. One of the main concerns is that I was unable to find the supplementary figures (Figures S1-S4).**

We apologize for this. We have made sure that supplementary figures are available in this reviewed manuscript.

**2. The explanation on how the stimuli was selected lacks detail. In the Stimuli section (l117 - l161) it is said that the tracks were sampled from a larger set. No detail is provided on the sampling method. Later on, in the discussion (l489) it is said “While these sets were assembled with the intention of maintaining uniformity in musical features”. This should be explained in the stimuli section. Moreover, some further analysis on how similar the music sets were (e.g.: Silhouette score) could better illustrate the picture.**

We thank the reviewer for highlighting this important issue. The sampling method for the work flow tracks is now explained in greater detail in the stimulus section of the methods (L146-152). We have incorporated the indicated quote from the methods, with further explanation:

*Work flow tracks were provided to us as .wav files by the music therapy app from the “anxious-to-energized” portion of their workflow playlist (sampling rate=44.1 KHz, bit depth=16). Out of the twelve tracks provided, four were selected for this study based on having musical features that were broadly similar and representative of the entire set. Deep focus and pop hits tracks were purchased and downloaded as .m4a files from Apple Music (sampling rate =44.1 kHz, bit rate =256 kbps). For both of these conditions, we shuffled each source playlist until the first sixteen tracks were each created by a different artist, and then selected these sixteen for inclusion.*

Following the reviewer’s suggestion, we have included the results of a clustering and silhouette analysis in Table S1, with a pointer in the main text (L173-175). This analysis applied K-means clustering (with K=2) to the musical feature data for the work flow and deep focus tracks in Table S1. The results show that all of the deep focus tracks group into a single cluster with a mean silhouette value of 0.95, indicating a (very) high degree of similarity in musical feature profile. Similarly, the work flow tracks all group into a single (different) cluster. However, their mean silhouette value was much lower at 0.55. Upon closer inspection, this lower value reflects a mixture of three well clustered tracks (with silhouette values of 0.78, 0.77, and 0.72) and one poorly clustered track (silhouette value = -0.08). Overall, these results indicate that the work flow and deep focus tracks examined here were generally well separated, with the exception of one work flow track, whose cluster membership was more ambiguous (i.e. close to 0). Increasing K to 3 reduced the mean silhouette value over the set (from 0.87 to 0.69) indicating that K=2 represents a better solution. In considering this analysis, we emphasize that there is no standard way of describing musical similarity/difference in featural terms. Some features are likely to weigh more heavily in perception than others, and there is also variance in perceptual weighting across individual listeners and listening conditions. It should also be noted that the correspondence between features as defined by MIR algorithms and perceptual “features” is not always clear. For these reasons, we now include audio of the work flow tracks as supplementary materials (S1-4 Audio.wav files). The other music tracks used in this study are all available commercially. This should allow any interested readers to hear these tracks for themselves and conduct their own analyses as they see fit.

*Explanations of each feature and how they were extracted are provided in Supplementary Material. See Table S1 for specific track names, arrangement of tracks into sets (for deep focus and pop hits), track-by-track break downs of musical features, and the results of a clustering/silhouette analysis indicating that the work flow and deep focus tracks are generally well separated into two different groups.*

**3. In the results for Group categorization (l324), it is stated: "Music genre preferences varied within groups but were comparable across them”. Here, it is not clear what the music genre preferences refer to or how they are comparable. The text references Table S2, which contains the Pleasure, Familiarity and Groove ratings. If this is what the text refers too, then this is explained in the discussion section, when Bayesian stats are presented (l513-l517). These results should be presented in the results section.**

We apologize for this misunderstanding. Table S2 was part of the supplementary materials that, due a technical glitch, were not available during the first review of this manuscript. This is Table S2:

***Table S2****. Preferred music genres in the four groups of participants. The number in each cell indicates the number of participants in that group that indicated that a particular genre was their favorite.*

| *Group* | *Rock* | *Pop* | *Classical* | *Rap* | *Jazz* | *Indie* | *Electronic* | *Metal* | *Country* | *R&B* | *Latin* | *Punk* |
| --- | --- | --- | --- | --- | --- | --- | --- | --- | --- | --- | --- | --- |
| *WF* | *14* | *10* | *7* | *7* | *2* | *3* | *1* | *1* | *4* | *1* | *0* | *0* |
| *Focus* | *14* | *9* | *6* | *6* | *7* | *1* | *2* | *3* | *1* | *0* | *1* | *0* |
| *Hits* | *20* | *10* | *10* | *2* | *2* | *2* | *1* | *1* | *0* | *0* | *0* | *0* |
| *Office* | *13* | *10* | *12* | *4* | *2* | *1* | *1* | *0* | *0* | *3* | *0* | *1* |

In addition to making sure that the supplementary materials are accessible, we have expanded the section regarding the genre preferences in the main text (L319-322):

*Among the four groups, the two most popular genres were Pop and Rock (see Table S2). An analysis of the genres selected as favorites by at least 10 participants—namely Rock, Pop, Classical, Rap, and Jazz—revealed no significant differences in their distribution across the groups (BF_01_=1081).*

**4. In the discussion, a main difference is stated between the “Work flow” condition and other conditions based on musical attributes of the music; namely, “musical features closely associated with perceived arousal, such as spectral flux” as well as strong rhythmic features such as pulse clarity. Another important difference is given by the “Work flow” sets being comprised of a single coherent track, while the other musical conditions (excluding “Office”) having multiple tracks with silence in between. I would argue that the entrainment allowed by a coherent continuous track could be part of the reasons for improved RT, or the cognitive load of adapting to a changing auditory environment a hindrance to the benefits of music.**

We thank the reviewer for bringing up this interesting perspective. We have included it in the updated discussion section (L509-512):

*It is plausible, for example, that entrainment facilitated by listening to a continuous, coherent track may have aided participants to perform the task correctly faster, whereas adapting to a dynamically changing auditory environment could have increased cognitive load and impaired performance.*

**### Minor comments**

**- p6, 155: "The office noise track was generated with the following 155 settings on the sound generator web page" - I would add an explanation on the criterion by which these settings were selected.**

We thank the reviewer for their suggestion. The following explanation has been added to the text (L159-160):

*The office noise track was generated with the following settings on the sound generator web page: “Room Tone” at 38% of maximum; “Air Co” at 50%, “Chatty Colleagues” at 89%, “Copy machine” at 66%; “Printing & Scanning” at 74%, “Office Noises” at 100%, “Keyboards & Mouse” at 58%, “Keyboards” at 73%, “Writing” at 68%; and “Office Clock” at 45% (these settings were determined by ear with the goal of simulating a typical open office environment; see supplementary materials audio S5* *for the recording used for this condition).*

Audio of the office noise track has been added as supplementary material so that readers can listen for themselves.

**- p7, 164: I would report MATLAB version used, as MIRToolbox has shown to work differently with different MATLAB versions.**

We thank the reviewer for catching this. The MATLAB version (R2022a) used is now reported.

**- p11, 267: "The same procedure was used..." - I am confused on which procedure this is as this is for only post-measured variables instead of pre-post (which is what the last analysis referred to). Is it the same analysis as the previous paragraph?**

We have clarified that sentence in the new version of the manuscript (L260-262):

*One-way between-participants ANOVAs with four levels (work flow, deep focus, pop hits, office noise) followed by post-hoc t-tests with Tukey correction were used to analyze self-reported musical familiarity, pleasure, and groove ratings made during the end survey.*

**- p17: "it also included the three-way interaction between audio condition, trial number, and DASS21 anxiety subscore." - Does this model also contain the previous terms with these variables (i.e.: AudioCondition*TrialNumber +DASS21)?**

We now specify the exact model that was tested (L441-443):

*Accordingly, using R, we computed a linear mixed model to predict RT that was the same as the best model described above (see also Table 2) except that it also included the three-way interaction between audio condition, trial number, and DASS21 anxiety subscore [i.e., FlankerCondition + AudioCondition*TrialNumber*DASS21Anxiety + DASS21Depression + DASS21Stress+ BMRQ + GOLD-MSI + (1|ID)].*

**- p20, 489: DB and CT introduced out of nowhere**

These initials correspond to author names, which we now clarify (L509):

*These guidelines were created by the music therapy app in consultation with co-authors D.B. and CT. D.B. and C.T. also reviewed each track in an iterative editorial process in which adherence to the acoustic guidelines was maintained.*

**Reviewer #2: General comments**

**In this study, the authors wanted to test if music advertised as able to improve work flow or to engage listeners in deep focus affects a cognitively demanding task (flanker test). They also tested if this music affected the listeners’ mood and state. They test this by running an online experiment on MTurk (n~200). The experiment consisted of a series of music, mental health, and mood-related questionnaires followed by a flanker task. This flanker task had to be solved while listening to three types of background music (work flow, deep focus, and pop hits) and a control condition (calm office noise). They found that the Work Flow music significantly improved mood (as measured by PANAS) and differentially decreased RTs with time on the flanker task. They did not find any effect on participants’ accuracy on the flanker task. The manuscript is clear in presenting the research question and all the relevant previous work in the area. The stimuli selection and the experimental procedure and clearly and thoroughly described in the manuscript. The experimental design and the data collected were consistent with the hypothesis they wanted to test. The statistical tools used were generally correct and well-interpreted by the authors.**

We thank the reviewer for their positive summary of our work.

**However, there are several points the authors should address for this manuscript to be ready for publication. The main concern is that the significant triple interaction (shown in Figure 6) seems to show a more negative slope of the RTs for the work flow condition but the predicted RTs are larger for most of the trials (~60 out of 72).**

We thank the reviewer for their thorough comments that have made our manuscript stronger. We address all the comments, including the one raised by the reviewer about Figure 6 in the following sections. We want to note, however, that Figure 6 does not stem from a triple interaction as indicated by the reviewer. We have clarified that in the updated manuscript to avoid any confusion (L451-452):

*To do this we computed a linear mixed model predicting RT as a function of fixed effects for trial number, total change in PANAS score, and their interaction, as well as a random intercept effect for participant [i.e., RT ~ TrialNumber*TotalPANAS + (1|ID)].*

**The authors properly disclosed their conflict of interest.**

Following the editor’s suggestion, we now further clarify that the conflict does not alter our adherence to PLoS One’s policies on data sharing:

*This work was funded by a grant from the company that provided the tracks for the work flow condition to Dr. Ripolles. Dr. Orpella has no competing interests. This does not alter our adherence to PLOS ONE policy on sharing data and materials.*

**Particular comments**

**P4-L85: Remove the “very” in “very different neural and behavioral responses” unless there is a quantification of this expectation.**

Updated as requested.

**P4-L94: I will not consider N=196 as a large-scale experiment. Please remove this adjective or add references that support this claim.**

Updated as requested.

**P5-L110: Why would the authors want to be able to detect a medium effect size with the given statistical power? Please provide a rationale for this decision. What was the dependent variable in your power analysis? The manuscript is full of statistical tests and it should be clear for which one they planned the power.**

We thank the reviewer for raising this issue. The dependent variable in our power analysis was the ability of the audio condition to change the mood of the participants. The reviewer is right that there are other statistical tests that we could have used to calculate the sample size. For example, we could have used the analysis of the effects of audio on the Flanker task which corresponds to a mixed between-within 4 x 4 repeated measures ANOVA with audio condition (work flow, deep focus, pop hits, office noise) as the between-subjects factor and flanker condition (congruent, incongruent, neutral, no-go) as the within-subjects factor. A power analysis using MorePower indicated a sample size of at least 22 participants per group for 80% power to find a medium effect size in this analysis. We chose the sample size required to study the effects of audio condition on mood (N=44) as it was higher than the one to study the effects of audio condition on Flanker accuracy (N=22). Note that calculating sample sizes for linear mixed models is not trivial and often requires simulating data. Here, we used the sample size calculation for the effect of audio condition on Flanker accuracy as a proxy for our analyses on RT. Regarding the size of the effect, previous research using a similar paradigm found medium to large effects of different audio conditions on pleasure and Flanker responses (Cloutier et al., 2020). In addition, previous research using a similar paradigm, also assessing different groups as we do here, and finding significant effects has used smaller sample sizes than the ones we employed (Cloutier et al., 2020; two groups, N=19 and N=21; Fernandez et al., 2019: two groups, N=19, N=33; Fernandez et al., 2021; two groups, N=15, N=15). We have updated the section regarding the sample size calculation accordingly (L102-107):

*A power analysis using MorePower (54) indicated a sample size of at least 44 participants per group for 80% power to find a medium effect size [partial eta^2^ = 0.06; (37)] in a between-subjects one-way ANOVA with four conditions (the analysis required to assess the effect of audio condition on mood). This statistical test was selected among other possible ones (e.g., effect of audio condition on Flanker accuracy) as it was the one requiring the largest sample size. In addition, previous research using a similar paradigm, assessing different groups as we do here, and finding significant effects has used smaller sample sizes than the one we employed [(37): two groups, N=19 and N=21; (38): two groups, N=19, N=33; (39): two groups, N=15, N=15].*

**P5-L110: How did the authors estimate the variability for the power analysis? Did they use data from a pilot study or an estimation based on previous studies? Please provide additional information on this matter to the manuscript.**

See our previous answer.

**P5-L131: Add a reference to support this affirmation: “Given prior research on the effects of listening to pop music during work, we expected this condition to negatively impact on-task performance.”**

We thank the reviewer for catching this omission. We have clarified this sentence and added relevant literature (L125-127):

*Given prior research on the effects of listening to popular music‒especially with lyrics‒during work and other complex tasks (58–60), we expected this condition to negatively impact on-task performance.*

**P7-L168: Supplementary material with details on how the musical features were extracted was not provided in the manuscript. Please add this document to the revised version.**

We apologize for the lack of supplemental materials in the original submission of this manuscript. All supplemental materials and figures are included in this reviewed version.

**P11-L271: ANOVAs assume that residuals are normally distributed, something that rarely happens on count data, especially if you experience any floor or ceiling effect. In the particular case of the data of this study, this assumption is not met mainly because the number of correct responses is not only a count variable but it is bounded between 0 and 18 with most of its mass (for most subjects) close to 18. The authors could explore models for bounded count data (generalized linear mixed-effect models for Poisson or Negative Binomial) to properly model the participant’s responses. A more direct approach could be to replicate the model structure of the RTs but using correct/incorrect as the dependent variable and “logit” as the link function of a generalized linear mixed effect model (although this seems to have convergence issues with your data). This could be the reason for the non-significant results on page 16 (L388 to L397).**

The reviewer brings forth an interesting point. As the reviewer points out, for most of participants the number of errors is minimal with accurate responses close to 18 (the maximum in each condition of the Flanker task). We agree with the reviewer that the data might be exhibiting a ceiling effect. It is thus possible that the lack of an effect of audio condition on Flanker accuracy is due to restricted variation in error rates. We have computed the analysis suggested by the reviewer, replicating the model structure of the RTs but using correct/incorrect as the dependent variable and the R function *glmer*. The interaction between audio condition and Flanker condition is still not-significant (p=0.17). We now report the mean and SD of the accuracy of the different conditions of the Flanker and have updated the limitation paragraph highlighting the ceiling effect as a possible explanation for the lack of effects of audio condition (L574-577):

*Indeed, most participants made few errors across the Flanker conditions (mean correct answers ± SD for all participants, regardless of audio condition: NoGo, 15.12 ± 4.93; Congruent, 17.66 ± 1.08; Incongruent, 16.65 ± 3.13; Neutral, 17.48 ± 1.63). This ceiling effect might account for the lack of significant effects of music background condition on Flanker accuracy*

**P11-L276: I do not think the substantial variability is a reason to use mixed-effect models. If you think that is the case please provide a quantifiable definition of substantial and references that justify the relationship with that and the recommendation to use linear mixed models.**

We apologize for not properly clarifying what we meant by variability in the data. We have updated that section clarifying the reason why we chose linear models and providing relevant citations (L269-273):

*Second, we also examined reaction times (RTs). RTs were only analyzed for correct trials. Given that participants may provide incorrect answers at different times (i.e., in different trials), missing data can pose a challenge in analyzing RTs, particularly when time is included as a fixed factor in the models (i.e., trial number). To address this issue, we opted for linear mixed models rather than ANOVAs for analyzing RTs (81, 82). We employed the lme4 package (83) within R (version 4.2.0) and RStudio (version 2022.02.2.485) for this analysis.*

**P13-L314: There is an extra “)” after “noise”.**

We thank the reviewer for catching this typo. It has been corrected.

**P13-L319: Figure S1 was not in the manuscript. Please add this document to the revised version.**

We apologize for the lack of supplemental materials in the original submission of this manuscript. All supplemental materials and figures are included in this reviewed version.

**P13-L320: A BF of 2.11 is usually not considered strong evidence for a given hypothesis. Please better explain the group differences in musical training.**

We provide further information and analysis regarding this matter (L315-318):

*For musical training the BF_01_ was 2.11. This lower BF is driven by a higher score displayed by the participants in the pop hits group (see Figure S1D). However, there was no significant effect of group of musical training (F(3,192)=2.38, p=0.07) with all post-hoc tests comparing each group score against each other showing no significant differences (all corrected ps>0.092).*

**P13-L324: Table S2 was not in the manuscript. Please add this document to the revised version.**

We apologize for the lack of supplemental materials in the original submission of this manuscript. All supplemental materials and figures are included in this reviewed version.

**P13-L327: Add the individual data points to Figure 3A.**

We thank the reviewer for this suggestion. Figure 3A has been updated accordingly:

**Figure 3.** *Effects of audio condition on mood.* **A.** Participants in the work flow (WF) condition exhibited greater improvements in mood than participants in the deep focus (Focus), pop hits (Hits), and office noise (Office) conditions (all ps<0.001, corrected for multiple comparisons). Bar plots show mean with standard deviation. **B.** Exploratory analysis indicating that mood improvement in the work flow condition was independent of individual variation in basal levels of anxiety as assessed with the DASS-21 (i.e., audio condition did not interact with the DASS-21 anxiety subscale). The same was true for the DASS-21 depression and stress subscales, but only results for anxiety are shown here. Solid lines show predicted values; dashed lines show 95% confidence intervals. For reference, DASS-21 Anxiety scores 10-13 are considered mild, 14-20 moderate, 21-27 severe, and 28+ extremely severe.

**P13-L327: It is not clear if the only statistically significant difference condition was work flow. I assume that when it is not mentioned the results were nonsignificant but please, if that is the case, make it explicit.**

We thank the reviewer for pointing out this lack of clarity. We now explicitly state that other comparisons were not significant (L328): *No other between-group comparisons were significant (all ps>0.080).*

**P13-L330: Clarify how you corrected for multiple comparisons when running the one-sample t-tests.**

We applied Bonferroni correction for 4 tests (p<0.0125). The section has been updated accordingly (L331):

*Furthermore, one-sample t-tests applied to each condition indicated that work flow was the only audio condition in which the total change in PANAS score from before to after the flanker task was significantly different than zero (work flow, t_49_=7.0, p<0.007, d=0.99, this p-value survives Bonferroni correction for the 4 tests computed; focus, t_49_=-0.879, p=0.38, d=0.124; pop hits, t_48_=-1.56, p=0.12, d=0.224; office noise, t_46_=-1.55, p=0.12, d=0.227).*

**P14-L355: Add the individual data points to Figure 4.**

We thank the reviewer for this suggestion. Figure 4 has been updated accordingly:

**Figure 4.** *End survey results.* Self-reported pleasure (**A**), groove (**B**), and familiarity (**C**) associated with the audio in the work flow (WF), deep focus (Focus), pop hits (Hits), and office noise (Office) conditions. Bars show mean with standard deviation. *** p<0.001, **p<0.05.

**P14-L361: When using null-hypothesis statistical testing the results are dichotomical: Differences are either different from zero (given a significance level set by the selection of alpha) or not. The authors should remove the “marginally less” statement from the text and the use of * for p=0.88 from the figure. This last issue is especially problematic since it could be mistaken by the more common use of * for p<0.05.**

The appropriate section and figure have been updated accordingly (L367-368):

*In addition, participants in the pop hits condition reported liking the audio less than those in the deep focus condition (t_89_=2.57, p_holm_=0.033, d=0.54), although the difference with the work flow condition was not significant (t_90_= 2.02, p_holm_=0.088, d=0.423).*

**P14-L369: How did the authors phrase the question about familiarity with the “office noise” condition?**

We thank the reviewer for catching this. We have specified the changes made when asking about office noise (L235-236): *For the office noise condition, we changed the word soundtrack for “audio”.*

**P16-L384: Figure S5 was not in the manuscript. Please add this document to the revised version.**

We apologize for the lack of supplemental materials in the original submission of this manuscript. All supplemental materials and figures are included in this reviewed version.

**P16-L388: Figure S2 was not in the manuscript. Please add this document to the revised version.**

We apologize for the lack of supplemental materials in the original submission of this manuscript. All supplemental materials and figures are included in this reviewed version.

**P17-L414: Figure S4 was not in the manuscript. Please add this document to the revised version.**

We apologize for the lack of supplemental materials in the original submission of this manuscript. All supplemental materials and figures are included in this reviewed version.

**P17-L418: Authors should provide a table with all the estimated parameters of the fitted model (with CIs). I would recommend they use the modelsummary R package.**

We thank the reviewer for this suggestion. We have use the modelsummary R package to generate confidence intervals. New Table S3 now displays the requested information (L405-406):

*Examining the best model [FlankerCondition + AudioCondition*TrialNumber + BMRQ + GOLD-MSI + DASS21 + (1|ID); see Table S3 for a full list of parameter estimates with 95% confidence intervals)], as determined by AIC comparisons, showed a main effect of flanker condition (χ²_2_=361.62, p < 0.001).*

Here is the new Table S3:

***Table S3****. Parameter estimates with 95% confidence intervals (CI) for the best model explaining RTs: FlankerCondition + AudioCondition*TrialNumber + BMRQTotal + GOLD-MSI + DASS21 + (1|ID). For flanker condition the baseline level is Neutral and for Audio it is Office Noise. Significant parameters are marked in bold.*

| ***Parameter*** | ***Estimate (CI)*** |
| --- | --- |
| ***Flanker Congruent*** | *-17.4 [-27.0, -7.8]* |
| ***Flanker Incongruent*** | *72.1 [62.3, 81.8]* |
| *Audio Focus* | *-17.3 [-104.1, 69.5]* |
| *Audio Pop Hits* | *-20.5 [-108.5, 67.5]* |
| *Audio Work Flow* | *-34.8 [-121.7, 52.2]* |
| *Trial Number* | *0.4 [0.0, 0.7]* |
| ***BMRQ Total*** | *-6.0 [-10.3, -1.8]* |
| *GOLD Training* | *145.6 [-27.0, 318.3]* |
| *DASS21 Depression* | *-4.0 [-9.1, 1.0]* |
| ***DASS21 Anxiety*** | *18.2 [11.8, 24.7]* |
| *DASS21 Stress* | *-2.7 [-9.8, 4.4]* |
| *Audio Focus * TrialNumber* | *0.3 [-0.3, 0.8]* |
| *Audio Pop Hits * TrialNumber* | *0.01 [-0.6, 0.5]* |
| ***Audio Work Flow * TrialNumber*** | *-0.77 [-1.3, -0.2]* |

**P17-L421: Please provide a p-value after “(slope; β=-0.421)”.**

We apologize for any misunderstanding. That value represents the estimated marginal trend for work flow (i.e., the estimated slope as calculated by emtrends before comparing it with the slope of other conditions). We have clarified this in the updated manuscript (L420-423):

*Post-hoc tests showed that participants in the work flow condition became faster over time, regardless of flanker condition with the rate of decrease in RT (**work flow slope: β=-0.421) being significantly steeper than in each of the other conditions (deep focus slope: β=0.615, difference in slope with work flow = 1.03, t_9959_=3.80, p_tukey_<0.001; pop hits slope: β=0.340, difference in slope with work flow = 0.76, t_9959_=2.79, p_tukey_=0.027; office slope: β=0.356, difference in slope with work flow = 0.77, t_9959_=2.83, p_tukey_=0.024).*

**P17-L423: The use of the wording “task performance” could be misleading since what the authors observed is a differential effect of the time on the response time but not on the accuracy of the responses. Please rephrase the last sentence of that paragraph.**

We have clarified what we mean by performance (L424-425):

*This indicated a significant effect of work flow music on performance (i.e., faster responses over time, with similar flanker accuracy).*

**P18-L425: A non-significant result could mean that there is no difference only if the sample size was determined with an apriori power analysis, otherwise this could attributed to a lack of statistical power. The non-significant three-way interaction does not “indicate” that the observed effect of work flow music on task performance over time was independent of anxiety severity as assessed by the DASS-21”.**

We have changed the word “indicates” for “suggests” (L444):

*The results showed that this three-way interaction was not significant (χ²3=3.01, p = 0.389). This suggests that the observed effect of work flow music on task performance over time was independent of anxiety severity as assessed by the DASS-21 (see Figure 5B), despite the overall slower performance of individuals with high levels of anxiety (see Figure S4A).*

**P18-L432: Authors should add a title to the color bar on Figure 6.**

We thank the reviewer for this suggestion. Figure 6 has been updated accordingly:

**Figure 6.** *Relationship between effects on mood and performance in the work flow condition.* Each line shows the model prediction for RT over time for a different change in total PANAS score (from -0.25 to 1). For participants who listened to work flow music, larger improvements in mood (black to blue gradient) were associated with increasingly rapid responses on flanker trials with increasing trial number (i.e., steeper slopes; p=0.0258).

**P18-L432: Two things in Figure 6 are interesting and not addressed in the manuscript: 1- Although larger PANAS scores are associated with a faster improvement of the RTs, it also seems to make the participants respond slower in the work flow condition for the first trials. 2- The predicted RTs cross around trial number 60, and trial number 72 the difference in RTs is quite small compared to the ones at the beginning of the experiment. The authors should explain both these effects in more detail.**

The reviewer brings forward an interesting point.

The linear predictions of these models do indeed suggest that greater mood improvement was associated with lower RTs at the start of the flanker task. One possible explanation for this is that participants who made greater mood improvements started in lower mood states, which could have negatively impacted their initial response speeds, in a similar way as basal anxiety levels appear to (see Fig. S4A). However, the analysis we present in Figure 6 is already exploratory (as noted explicitly on L466), which makes us hesitant to engage in further interpretation in the main text, which feels overly speculative.

Nevertheless, in attempt to address the reviewer’s point empirically, we have tried to test the possibility that participants with higher basal anxiety (and thus slower overall flanker task RTs) were also more likely to benefit from listening in the work flow condition. In other words, if the work flow music was particularly helpful to those who started in a worse place emotionally—e.g., because they have more room to improve—this might help explain why the model predicts slower starting RTs for participants who improved the most. Accordingly, we reran the model depicted in Figure 6 but included a triple interaction between basal anxiety, mood benefit, and trial number [i.e., RT ~ TrialNumber*TotalPANAS*DASS21Anxiety+(1|ID)]. This triple interaction was not significant (p=0.1042368). This suggests that either: (1) the dependence of lower RTs on the interaction between trial number and mood benefit is unlikely to be driven by higher basal anxiety; or (2) that this analysis is underpowered and shouldn’t be used to support any further conclusions. Given the exploratory nature of the main analysis in Figure 6, we prefer to not include these further ideas or analyses in the main text. However, if the reviewer really thinks that it is critical for the correct interpretation of the paper we will include them in the next review.

**P18-L447: If I am not mistaken, the authors could not affirm that “These mood effects were not explained by differences between audio conditions in self-reported musical pleasure” since there is a main effect of pleasure on PANAS change. I assume the previous statements refer to the interaction being non-significant, but given that the mean pleasure level is not the same for all the musical stimuli, music's effect on mood could, in fact, be partially mediated by pleasure.**

We have clarified this sentence (L468-470):

*While there was not a significant interaction between audio condition and pleasure, there was a main effect of pleasure on total change in PANAS score with greater improvements in mood being associated with greater music liking across conditions*
