# Supplementary material for: Effects of music advertised to support focus on mood and processing speed
Source: PLoS One. 2025 Feb 12;20(2):e0316047. doi: 10.1371/journal.pone.0316047 (PMC11819607; doi:10.1371/journal.pone.0316047)
Supplement: Attachment — Submitted filename: 01_AnswerToReviewers_final.docx [file pone.0316047.s011.docx]

We thank the two anonymous reviewers for their comments. Below we provide detailed answers to the remaining issues raised by Reviewer 2. Where possible, we have included the edited sections after each comment, and all edited text is printed in blue in our revised manuscript.

**Reviewer #1**

**All comments have been properly addressed. The missing supplementary material is sound, complementing the main text. The clustering analysis performed on the selected music is satisfactory to understand that the sets in the "deep focus" playlist are similar with each other. This is similar for the sets in the "work flow" playlist, with the exception of one track, which is still better clustered with the "work flow" set.**

We thank the reviewer for their positive feedback on the reviewed version of the manuscript. We thank the reviewer for their comments on the previous version of the manuscript, which we believe have made the paper stronger.

**Reviewer #2**

**The authors have thoroughly addressed all comments, concerns, and suggestions. They accepted the majority and, in cases where they did not, provided a sufficiently robust rationale.**

We thank the reviewer for their positive feedback on the reviewed version of the manuscript.

**However, I believe there are still two points that require further clarification:**

**1- The rationale for fitting mixed-effects models remains unclear. Random effects should only be included when residuals are correlated, typically due to the way units are sampled (i.e., randomly). I recommend that the authors either justify the use of mixed-effects models appropriately or refrain from providing a justification if it cannot be done accurately.**

Following the reviewer’s advice, we have removed the justification for using linear mixed-effects models. The section in the Methods has been updated accordingly:

*Page 11, lines 269-271*

*Second, we also examined reaction times (RTs). RTs were only analyzed for correct trials. We opted for linear mixed models rather than ANOVAs for analyzing RTs (81,82). We employed the lme4 package* *(83) within R (version 4.2.0) and RStudio (version 2022.02.2.485) for this analysis.*

**2- The model and analysis of the triple interaction (Figure 6) should be included as supplementary material, with a reference to it added in the main text.**

We thank the reviewer for their suggestion. We have added a table to the supplementary material describing the main results of Figure 6 and we have referenced it in the main text:

*Page 18, lines 449-450*

*The results (see Table S4 for a full list of parameter estimates with 95% confidence intervals) showed that the interaction term was significant (χ²_1_=4.96, p=0.0258),*

*Supplementary material*

***Table S4****. Parameter estimates with 95% confidence intervals (CI) for the exploratory model assessing the relationship between RTs and change in mood for the Work Flow group (see Figure 6 in the main text): RT ~ TrialNumber*TotalPANAS+ (1|ID). Significant parameters are marked in bold.*

| ***Parameter*** | ***Estimate (CI)*** |
| --- | --- |
| *Trial Number* | *0.0 [-0.5, 0.4]* |
| *Total PANAS* | *49.4 [-97.0, 195.8]* |
| ***TrialNumber * Total PANAS*** | *-0.8 [-1.6, -0.1]* |
